# Social Disparities in Exposure to Point-of-Sale Cigarette Marketing

**DOI:** 10.3390/ijerph13121263

**Published:** 2016-12-21

**Authors:** Mohammad Siahpush, Paraskevi A. Farazi, Jungyoon Kim, Tzeyu L. Michaud, Aaron M. Yoder, Ghada Soliman, Melissa K. Tibbits, Minh N. Nguyen, Raees A. Shaikh

**Affiliations:** 1 College of Public Health, University of Nebraska Medical Center, 984365 Nebraska Medical Center, Omaha, NE 68198, USA; msiahpush@unmc.edu (M.S.); evi.farazi@unmc.edu (P.A.F.); jungyoon.kim@unmc.edu (J.K.); tzeyu.michaud@unmc.edu (T.L.M.); aaron.yoder@unmc.edu (A.M.Y.); ghada.soliman@unmc.edu (G.S.); mtibbits@unmc.edu (M.K.T.); 2 College of Public Health, University of Oklahoma Health Sciences Center, 801 N.E. 13th Street, Oklahoma City, OK 73104, USA; raees-shaikh@ouhsc.edu

**Keywords:** point-of-sale cigarette marketing, social disparities, income, race/ethnicity

## Abstract

While most ecological studies have shown that higher levels of point-of-sale (POS) cigarette marketing are associated with larger proportions of residents from lower socioeconomic and minority backgrounds in neighborhoods, there are no studies that examine individual-level social disparities in exposure to POS cigarette marketing among smokers in the United States. Our aim was to examine these disparities in a Midwestern metropolitan area in the United States. We conducted a telephone survey to collect data on 999 smokers. Cigarette marketing was measured by asking respondents three questions about noticing advertisements, promotions, and displays of cigarettes within their respective neighborhoods. The questions were combined to create a summated scale. We estimated ordered logistic regression models to examine the association of sociodemographic variables with exposure to POS cigarette marketing. Adjusted results showed that having a lower income (*p* < 0.003) and belonging to a race/ethnicity other than “non-Hispanic White” (*p* = 0.011) were associated with higher levels of exposure to POS cigarette marketing. The results highlight social disparities in exposure to POS cigarette marketing in the United States, which can potentially be eliminated by banning all forms of cigarette marketing.

## 1. Introduction

Although cigarette smoking causes an estimated 480,000 deaths in the United States and more than 16 million people in the country suffer from smoking-related diseases each year [1], tobacco remains one of the most heavily marketed products [2]. In 2013, tobacco companies spent $8.9 billion on cigarette marketing. About 89% of this expenditure was made at the point of sale (POS) [3] in the following three marketing areas: cigarette pack displays, advertisements, and promotional and price incentives for consumers [4,5].

A focus of the research pertaining to POS cigarette marketing has been on examining the association of the amount of POS cigarette marketing in geographically defined areas such as communities or neighborhoods and the sociodemographic composition of those areas. For example, Barbeau et al. examined six communities in Boston, Massachusetts, and found that higher levels of POS marketing were associated with higher proportions of residents from lower socioeconomic and non-white backgrounds [6]. Similarly, Law et al. studied 10 business districts in Eastern Massachusetts and reported that a higher proportion of businesses displaying cigarette advertisements was associated with a lower per capita income and a higher proportion of minorities [7]. In a different study, Siahpush et al. collected POS cigarette marketing data from all the stores that were licensed to sell tobacco in 84 randomly selected neighborhoods in a Midwestern metropolitan area and found that marketing was considerably more common in neighborhoods with a lower average income [8]. However, they did not find evidence for an association between marketing and racial/ethnic composition of the neighborhoods. 

All previous studies examining the association between POS cigarette marketing and sociodemographic factors in the United States share two common characteristics. First, they are all ecological; i.e., they use a geographic area as the level of analysis and compare the amount of POS cigarette marketing in areas with varying sociodemographic compositions. Ecological studies describe groups of individuals rather than the individuals themselves [9]. Such studies are subject to the “ecological fallacy”, which refers to incorrectly making inferences about individuals from ecological studies [10,11,12], or more generally, mistakenly using observed associations between variables at one aggregation level as evidence for associations at a different aggregation level [13]. Thus, the fact that there is more POS cigarette marketing in areas with a higher proportion of individuals from more disadvantaged backgrounds does not necessarily mean that all the individuals from such backgrounds are exposed to a higher level of POS cigarette marketing. Second, previous studies have measured sociodemographic composition for the entire population of each area and made no distinction between smokers and non-smokers. Examining exposure specifically among smokers is important because POS cigarette marketing has been shown in recent research to be related to cravings to smoke, the urge to buy cigarettes, unplanned purchases of cigarettes, and smoking cessation among smokers [14,15,16]. To our knowledge, there are no published studies that examine the association of exposure to POS tobacco marketing and sociodemographic variables among smokers at the individual level in the United States. The aim of our study was to examine social disparities in exposure to POS cigarette marketing by assessing the individual-level association of exposure to POS cigarette marketing with income, education, and race/ethnicity in a sample of smokers in Omaha Metropolitan Area, Nebraska, in the United States.

## 2. Materials and Methods 

### 2.1. Sample

A total of 999 adult respondents were recruited in Omaha, Nebraska, using random digit dialing (47.2%) and placement of local advertisements (52.8%) in media such as the major daily newspaper and advertising website Craigslist, in 2014. All data were collected using structured telephone interviews. Respondents eligible for the study spoke English, were 18 years of age or older, were current smokers, meaning that they had smoked more than 100 cigarettes in their life [17], and smoked five or more cigarettes a day at the time of recruitment. Those who responded “never” to the following question were excluded from the study: “How often do you visit the stores in the neighborhood where you live? By stores, we mean such places as convenience stores, gas stations, grocery stores, supermarkets, drug stores, liquor stores, and tobacco stores.” Response options were 1 = never, 2 = sometimes, 3 = frequently, and 4 = always. More detail about the sample and design is provided elsewhere [14,15,16,18]. Ethics approval for the study protocol was obtained from the University of Nebraska Medical Center Institutional Review Board. Informed consent was obtained from each participant verbally as the data collection was done through telephone interviews. 

While the study sample was not a random sample, its sociodemographic composition was similar to the subsample of smokers in the center city of Nebraska Metropolitan Statistical Areas (i.e., approximately Omaha) in the Behavioral Risk Factor Surveillance System (BRFSS) [19]. For example, the gender distribution in the entire sample and that of the BRFSS were identical. The mean age was 47.8 years in our sample and 53 years in the BRFSS. The percentages of non-Hispanic Whites, non-Hispanic Blacks, and Hispanics in our sample were 65.9, 24.2, and 3.1, respectively, and 86.3, 9.1, and 1.5, respectively, in the BRFSS. The percentage of respondents with a high school diploma or a lower level of education was 49.9 in our sample and 46.3 in the BRFSS. The median income was $22,500 in our sample and $30,000 in the BRFSS.

### 2.2. Measurement

To measure the outcome, i.e., exposure to POS cigarette marketing, the survey asked each respondent the following three questions: “When you are in a store in your neighborhood, how often do you notice tobacco ads?”; “When you are in a store in your neighborhood, how often do you notice tobacco promotions such as special prices, multi-pack discounts, or free gift with purchase of cigarettes?”; and “When you are in a store in your neighborhood, how often do you notice cigarette pack displays?” [14,15,16,18]. Possible responses to each question were: 1 = never, 2 = rarely, 3 = sometimes, 4 = often, and 5 = always. The responses to the three questions were summed to create a scale of exposure to POS tobacco marketing with scores ranging from 3 (low marketing) to 15 (high marketing) [14,15,16] and a Cronbach’s alpha of 0.64. This summative scale has been used in previous research and is predictive of urges to buy cigarettes, impulse purchases of cigarettes, and smoking cessation [14,16]. 

The following explanatory variables were included in the analysis: annual household income, education, race/ethnicity, sex, age, frequency of visits to stores, and method of recruitment. Respondents were asked, “Is your annual household income from all sources …?” and provided with the following income categories: less than $15,000; $15,000 to less than $20,000; $20,000 to less than $25,000; $25,000 to less than $35,000; $35,000 to less than $50,000; $50,000 to less than $75,000; and $75,000 or more. The midpoint of income categories was used as the income of each respondent. For example, we used $30,000 for an individual who reported an income of $25,000 to less than $35,000. Due to the relatively low frequencies in the last two categories, we combined them and assumed their midpoint to be $75,000. 

Education was measured using the question, “What is the highest grade or year of school you completed?” and divided into the following groups: less than high school, high school graduate, some college, and college graduate. Race/ethnicity was categorized as non-Hispanic White, non-Hispanic Black, Hispanic, and other based on the following two questions: “Are you Hispanic or Latino?” and “Which one or more of the following would you say is your race? White, Black or African American, Asian, Native Hawaiian or Other Pacific Islander, American Indian or Alaska Native, and other.” Age was measured using the question “What is your age?” Method of recruitment was dichotomized into “random digit dialing” versus “other.”

### 2.3. Statistical Analysis

In all analyses, we omitted observations that had a missing value for any of the analysis variables. This constituted 4.9% of the sample. Only 0.5% of observations had a missing value for the outcome. Income had the highest percentage of missing observations (4.2%). The sample size for the final analysis was 950. We used ordered logistic regression to model the effect of covariates on exposure to POS cigarette marketing. Covariates whose *p*-values were greater than 0.1 in the bivariate models were not included in the multivariable model. 

## 3. Results

Table 1 shows the characteristics of the sample. The mean of the scale of exposure to POS cigarette marketing was 8.99 (standard deviation: 3.32; range: 3–15). Mean income was $31,760 and 49.89% of the sample had an education at or below high school level. Participants who were non-Hispanic White comprised 65.89% of the sample. The percentages of participants who were 18–24, 25–39, 40–45, and over 55 years old were 7.94, 20.97, 36.97, and 34.11, respectively. The percentage of respondents reporting that they always visited stores in their neighborhoods was 51.69. Those recruited through random digit dialing consisted of 45.23% of the sample.

Table 2 provides unadjusted and adjusted odds ratios for the association of covariates with exposure to POS cigarette marketing. In the unadjusted analyses, all covariates had a *p*-value less than 0.1. Therefore, they were all included in the multivariable regression. The adjusted results showed overwhelming evidence that higher income was associated with a lower probability of increased exposure to POS marketing (*p* = 0.003). Education was not associated with the outcome (*p* = 0.179). There was some evidence that race/ethnicity was associated with exposure to POS cigarette marketing (*p* = 0.011) such that Hispanics had the highest probability of increased exposure followed by Non-Hispanic Blacks, non-Hispanic Whites, and “other”. There was strong evidence that males had a higher probability of increased exposure than females (*p* < 0.001). Younger age was associated with a higher probability of increased exposure (*p* < 0.001). Higher frequency of visiting stores (*p* < 0.001) and recruitment via a method other than random digit dialing (*p* < 0.001) were associated with a higher probability of increased exposure to POS cigarette marketing. 

In supplementary analyses, we examined the association of exposure to POS cigarette marketing with sociodemographic composition of the zip codes of the respondents’ place of residence (see Table S1 in Supplementary Materials). We found no evidence of an association with household median income (*p* = 0.893), poverty rate (*p* = 0.868), percent with less than high school education (*p* = 0.445), and percent non-white (*p* = 0.115).

## 4. Discussion

We used data from a sample of smokers in Omaha Metropolitan Area, Nebraska, in the United States and found social disparities in exposure to POS cigarette marketing. Smokers with lower income and from non-white backgrounds were exposed to higher levels of marketing. Furthermore, being male or younger was associated with higher exposure to marketing.

By focusing on an individual-level analysis which includes common sociodemographic variables, this study improves our understanding of disparities in exposure to POS cigarette marketing. Our findings regarding income and racial disparities in exposure to POS cigarette marketing at the individual level were consistent with previous ecological studies in the United States [6,7,8]. However, while our finding that non-Hispanic Blacks and Hispanics were exposed to higher levels of marketing was consistent with some previous ecological studies [6,7], it was not consistent with an ecological study conducted in the same geographic region about five years prior to the current study. In that study, Siahpush et al. found no association between total amount of cigarette marketing and percentages of African American or Hispanic in neighborhoods of the Omaha Metropolitan area [8]. This apparent inconsistency may be due to the fact that conclusions from ecological analyses cannot be extrapolated to the individual level and that Siahpush et al.’s study examined racial and ethnic distribution of the population in each neighborhood as a whole instead of only examining the subpopulation of smokers in those neighborhoods. 

Our findings that male and younger smokers were exposed to higher levels of marketing were consistent with the results of a study of the general population in Bangladesh, Thailand, and Uruguay [20] and studies of the population of smokers in India, Malaysia, and China [21,22,23]. 

The finding that smokers recruited through local advertisements in newspapers or Craigslist were more likely to report a higher level of exposure to POS marketing than those recruited through random digit dialing is possibly due to the fact that the former group pays more attention to printed or written material, including signage for cigarette marketing in stores.

The results of this study reflect marketing efforts of the tobacco industry. In fact, analyses of tobacco industry documents show that it has a long history of targeting lower socioeconomic individuals, minorities, and youth [1,24,25,26,27]. There are several examples of such efforts. In the 1970s, Reynolds R. J. developed a program to provide discount cigarette coupons to inner-city low-income African Americans [28]. In the 1990s, Philip Morris debuted a magazine called Unlimited which targeted young adults and featured elements of the Marlboro brand identity in both advertising and content [29]. In the same decade, Reynolds R. J.’s efforts in expanding their urban marketing included directly targeting the homeless by advertising cheaper brands to “street people” [30]. In the 2000s, Brown and Williamson placed a greater number of signs in stores in communities with predominantly low-income and African American individuals [31]. To appeal to young African Americans, Brown and Williams used strategies such as using urban culture and language to promote menthol cigarettes and sponsoring hip-hop bar nights with samples of menthol cigarettes [31,32].

A weakness of the current research is the potentially low generalizability of the results to other regions or countries due to the fact that the sample was drawn from a Midwestern metropolitan area in the United States. Another weakness of the study pertains to the subjective measurement of POS cigarette marketing. We asked respondents to report on how often they noticed POS cigarette marketing. This poses a problem with recall bias, which may be true for all measures that rely on the memory of the individual to provide a report of an observation that occurred in the past. Additionally, our subjective measure of POS cigarette marketing may not be an accurate measure of the actual or objective level of exposure to marketing. An objective measure would provide an audit of cigarette marketing in stores that the individual visits during a given time period. Examining the actual amount of marketing is important because conscious recognition of marketing is not the only influence on consumer decisions and behaviors; environmental factors that are not consciously perceived by the individual can lead to decision processes that take place entirely outside of awareness [33,34,35,36]. Finally, the subjective nature of the measurement of POS cigarette marketing warrants mentioning a caveat related to the observed association between lower income and higher exposure to POS marketing. This association may not be due to an actual difference in the amount of POS cigarette marketing that lower income smokers encounter compared to other smokers. The reason for the association may instead be due to lower income smokers being more likely to seek out price promotions and thus notice them more often than other smokers. 

## 5. Conclusions

We found notable social disparities in exposure to POS cigarette marketing such that lower income smokers and those from minority backgrounds were exposed to higher levels of marketing. Our findings, coupled with previous reports that exposure to POS cigarette marketing can act as a barrier to smoking cessation among adults [16,37] and promotes smoking among youth [38,39], indicate that policies aimed at reducing disparities in exposure to marketing may help reduce existing disparities in smoking prevalence. Banning all forms of tobacco marketing, which has been shown to reduce smoking consumption and prevalence [40,41,42,43], as has been recommended by the Framework Convention on Tobacco Control [44] and implemented by countries such as Australia, Canada, Norway, Ireland, Finland, Iceland, Croatia, and Thailand can be a critical step towards eradicating social disparities in exposure to cigarette marketing.

## Figures and Tables

**Table 1:** Sample characteristics (*n* = 950).

Variables	% or Mean (Standard Deviation)
Exposure to point of sale marketing	8.99 (3.32)
Annual household Income ($1000)	31.76 (25.83)
Education	
Less than high school	10.17
High school graduate	39.72
Some college	36.86
College graduate	13.24
Race/ethnicity	
Non-Hispanic White	65.89
Non-Hispanic Black	24.15
Hispanic	3.07
Other	6.89
Sex	
Male	42.48
Female	57.52
Age	
18–24	7.94
25–39	20.97
40–54	36.97
55+	34.11
Frequency of visits to stores	
Sometimes	11.65
Frequently	36.65
Always	51.69
Method of recruitment	
Random digit dialing	45.23
Other	54.77

**Table 2:** Odds ratios (OR) and 95% confidence intervals (CI) from ordered logistic regression of POS cigarette marketing on covariates (*n* = 950).

Variables	Unadjusted ^a^ OR (95% CI)	*p*-Value	Adjusted ^b^ OR (95% CI)	*p*-Value
Income ($1000)	0.98 (0.98–0.99)	<0.001	0.99 (0.98–1.00)	0.003
Education		<0.001		0.179
Less than high school	1.00		1.00	
High school graduate	1.00 (0.68–1.47)		1.24 (0.84–1.83)	
Some college	0.84 (0.57–1.23)		1.20 (0.81–1.78)	
College graduate	0.40 (0.25–0.63)		0.84 (0.51–1.38)	
Race/ethnicity		<0.001		0.011
Non-Hispanic White	1.00		1.00	
Non-Hispanic Black	2.16 (1.65–2.83)		1.56 (1.17–2.08)	
Hispanic	2.57 (1.37–4.81)		1.70 (0.88–3.29)	
Other	1.26 (0.81–1.96)		0.96 (0.62–1.50)	
Sex		<0.001		<0.001
Male	1.58 (1.26–1.97)		1.57 (1.24–1.98)	
Female	1.00		1.00	
Age		<0.001		<0.001
18–24	1.00		1.00	
25–39	0.57 (0.36–0.89)		0.59 (0.37–0.94)	
40–54	0.36 (0.23–0.54)		0.45 (0.29–0.70)	
55+	0.19 (0.12–0.29)		0.30 (0.19–0.47)	
Frequency of visits to stores		<0.001		<0.001
Sometimes	1.00		1.00	
Frequently	2.27 (1.53–3.36)		1.90 (1.28–2.84)	
Always	2.99 (2.04–4.39)		2.49 (1.68–3.68)	
Method recruitment		<0.001		<0.001
Random digit dialing	0.36 (0.28–0.45)		0.64 (0.49–0.82)	
Other	1.00		1.00	

^a^ From bivariate result. ^b^ Adjusted for the effect of all covariates.

## Supplementary Materials

The following are available online at www.mdpi.com/1660-4601/13/12/1263/s1, Table S1: Odds ratios (OR) and 95% confidence intervals (CI) from ordered logistic regression of POS cigarette marketing on area-level sociodemographic factors.
