# Supplementary material for: Social Disparities in Exposure to Point-of-Sale Cigarette Marketing
Source: Int J Environ Res Public Health. 2016 Dec 21;13(12):1263. doi: 10.3390/ijerph13121263 (PMC5201404; doi:10.3390/ijerph13121263)
Supplement: Supplementary file 1 [file ijerph-13-01263-s001.pdf]

Supplementary Materials: Social Disparities in Exposure to Point-of-Sale Cigarette Marketing

Mohammad Siahpush, Paraskevi A. Farazi, Jungyoon Kim, Tzeyu L. Michaud, Aaron M. Yoder, Ghada Soliman, Melissa K. Tibbits, Minh N. Nguyen and Raees A. Shaikh

Table S1. Odds ratios (OR) and 95% confidence intervals (CI) from ordered logistic regression of point-of-sale (POS) cigarette marketing on area-level sociodemographic factors.

Variables	Adjusted [a] OR (95% CI)	*p*-Value
Median household income	1.00 (1.00–1.00)	0.893
Poverty rate	1.00 (0.99–1.01)	0.868
Percent with less than high school education	1.00 (0.99–1.02)	0.445
Percent non-white	1.00 (1.00–1.01)	0.115

[a] Adjusted for the effect of individual-level variables including income, education, race/ethnicity, sex, age, frequency of visits to stores, and method of recruitment.

© 2016 by the authors. Submitted for possible open access publication under the terms and conditions of the Creative Commons Attribution (CC-BY) license (http://creativecommons.org/licenses/by/4.0/).
